# Cytokine-induced killer cell therapy for advanced pancreatic adenocarcinoma: A case report and review of the literature

**DOI:** 10.3892/ol.2013.1200

**Published:** 2013-02-19

**Authors:** WEI LI, LIN PING XU, LING DI ZHAO, LI WANG, YONG ZHANG, QUAN LI GAO, LING MAI

**Affiliations:** 1 Department of Biotherapy, The Affiliated Tumor Hospital of Zhengzhou University, Henan Cancer Hospital, Zhengzhou 450003, P.R. China; 2 Department of Science Research, Henan Cancer Hospital, Zhengzhou 450003, P.R. China; 3 Institute of Tumor Research, Henan Cancer Hospital, Zhengzhou 450003, P.R. China

**Keywords:** pancreatic adenocarcinoma, cytokine-induced killer cells, advanced

## Abstract

Patients with advanced pancreatic adenocarcinoma have a poor prognosis, and to date, no treatment method has had a significant impact on the disease. In general, the mean overall survival time of such patients receiving conventional chemotherapy and radiotherapy is <6 months. In the present case report, a patient with advanced pancreatic adenocarcinoma experienced a longer progression-free survival (PFS) of >19 months, following cytokine-induced killer (CIK) cell therapy. To the best of our knowledge, no study has previously described such a beneficial effect on patients only receiving CIK cell immunotherapy. Based on these findings, CIK cell therapy may be a potential treatment regimen that is capable of leading to an improved prognosis in certain patients with advanced pancreatic adenocarcinoma.

## Introduction

The typical characteristics of pancreatic adenocarcinoma are its early local recurrence, systemic dissemination and poor response to chemotherapy and/or radiotherapy, thus rendering a poor prognosis with currently used treatment methods. The lack of traditional therapeutic options has motivated the search for novel immunotherapeutic approaches to pancreatic cancer, including cellular immunotherapy (1,2). At present, despite the development of chemotherapy drugs, research concerned with advanced pancreatic adenocarcinoma has only made modest progress, and the prognosis of advanced pancreatic adenocarcinoma remains extremely poor (3–5). Gemcitabine-based regimens are typically offered as standard approaches of care, and the majority of patients succumb within 6 months (6). The majority of elderly patients who undergo surgical resection are not able to tolerate the side-effects of chemotherapy and/or radiotherapy following disease relapse. Celluar immunotherapy has become the fourth treatment method for malignant tumors (7–10), which may be a promising approach for these patients. Cytokine-induced killer (CIK) cells are considered to be antitumor effector cells that are capable of rapid proliferation *in vitro*, with stronger antitumor activity and a broader spectrum of tumor targets than other described antitumor effector cells (10). Moreover, CIK cells are able to regulate and generally enhance immune function in cancer patients (11). There are limited data concerning adoptive immunotherapy in pancreatic adenocarcinoma. In the present study, we describe a patient with advanced pancreatic adenocarcinoma, who experienced a longer progression-free survival time (PFS) of >19 months, following administration of CIK cell immunotherapy. The study was approved by the Ethics Committee of Zhengzhou University, Zhengzhou, China. Written informed consent was obtained from the patient.

## Case report

**Figure 1:**
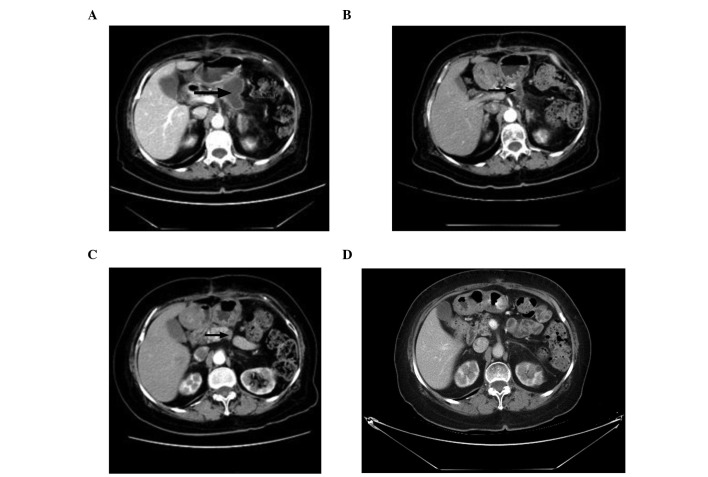
(A) November 10, 2010: Postoperative arterial phase contrast-enhanced computed tomography (CT) scan showing a low-density nodule. (B) February 14, 2011: Following 4 cycles of CIK cell infusion, the arterial phase contrast-enhanced CT scan shows that the low-density nodule has markedly decreased in size. (C) July 11, 2011: Following 12 cycles of CIK cell infusion, the arterial phase contrast-enhanced CT scan reveals that the low-density nodule has slighlty decreased in size. (D) February 29, 2012: The CT scan shows that the low-density nodule has almost completely disappeared.

A 77-year-old female was admitted to Henan Cancer Hospital, China, with intermittent abdominal pain for one month. Following investigation, pancreatic cancer was suspected by the surgeons. On October 14, 2010, the patient underwent resection of the pancreatic body and tail and the spleen. Analysis of the pancreatic mass revealed poorly differentiated adenocarcinoma, including squamous cell carcinoma differentiation, and the bilateral margins were positive. Abdominal contrast-enhanced computed tomography (CT) reexamination after one month revealed that a low-density nodule had emerged at the resection margin (Fig. 1A). Cancer- and tissue-specific markers tested, including carbohydrate antigen-199 (CA-199), carbohydrate antigen-724 (CA-724) and carcinoembryonic antigen (CEA), were observed to be elevated. Notably, the CA-199 level had risen to 1,000 U/ml (reference range, 0–35 U/ml). Considering that the patient demonstrated poor health and low Karnofsky performance status (KPS) scores (50 scores), as well as being unable to tolerate the side-effects of chemotherapy and/or radiotherapy, CIK cell immunotherapy was administered. Mononuclear cells were collected from the patient’s 50 ml peripheral blood and cultured in GT-T551 medium containing anti-CD3 antibody, recombinant human interleukin-1α (IL-1α) and interferon-γ (IFN-γ), at 37°C with 5% CO_2_ for 24 h. Subsequently, recombinant human IL-2 (rhIL-2) was added to the medium. The medium was replaced by fresh IL-2- and IFN-γ-containing medium every 5 days. At day 10, CIK cells were harvested and their phenotypes were analyzed. All products were free of bacterial, mycoplasma and fungal contamination. The endotoxin level was <5 EU. Phenotypic analysis of autologous CIK cells in the patient prior to culture and following 10 days of cultivation demonstrated that the percentages of CD3^+^, CD3^+^CD4^+^, CD3^+^CD8^+^, CD3^+^CD56^+^ and CD25^+^ cell subsets increased from 45.40±5.21, 29.08±4.86, 18.80±5.45, 3.77±1.665 and 12.51±4.01% to 90.06±9.22, 44.50±8.18, 38.40±4.19, 15.21±4.53 and 31.75±5.87%, respectively (P<0.01). However, the percentages of CD3^+^/16^+^/56^+^, CD14^+^ and CD20^+^ cell subsets decreased from 14.73±3.54, 16.46±6.06 and 11.19±3.18% to 6.78±1.91, 5.87±2.09 and 7.84±2.53%, respectively (P<0.05). The total number of CIK cells in one cycle is ∼ 5×10^9^. From November 10, 2010 to June 27, 2011, the patient recieved 4 cycles of CIK cell immunotherapy and 2 million units of IL-2 from day 1–5 per infusion of CIK cells. In the period of CIK cell therapy, no adverse reactions were observed. Following 4 cycles of CIK cell immunotherapy, the abdominal contrast-enhanced CT reexamination demonstrated that the low-density nodule reduced significantly (Fig. 1B). The cancer- and tissue-specific markers (CA-199, CA-724 and CEA) returned to their normal levels. The patient then received another 12 cycles of CIK cell immunotherapy. Abdominal contrast-enhanced CT examination indicated that the low-density nodule had slightly decreased (Fig. 1C) compared with the result demonstrated in Fig. 1B. The cancer- and tissue-specific marker levels (CA-199, CA-724 and CEA) remained at their normal levels. Following 16 cycles of CIK cell immunotherapy, it was recommended that the patient undergo abdominal contrast-enhanced CT examination every 3 months. The abdominal CT scan on February 29, 2012 demonstrated that the low-density nodule had almost disappeared (Fig. 1D). From March 7, 2012 to April 29, 2012, the patient received a further 4 cycles of CIK cell infusion to ensure treatment efficacy. However, on May 28, 2012, the abdominal contrast-enhanced CT scan revealed that the low-density nodule had emerged in the same place as previously, and the CA-199 level had elevated to 1,000 U/ml once more. At present, the progression-free survival time (PFS) of the patient is >19 months and the patient has not succumbed. During immunotherapy, the patient had a good quality of life and their KPS score increased from 50 to 80.

## Discussion

In the field of oncology, pancreatic cancer remains a fatal disease with a poor prognosis, thus presenting a challenge for oncologists (12,13). In patients with advanced pancreatic cancer, the mean overall survival time is <6 months (6). Chemotherapy and radiotherapy are the most common approaches for treating locally relapsed and metastatic pancreatic cancer (14); however, the overall survival time is only modestly prolonged. Additionally, adverse reactions to chemotherapy/radiotherapy may be not tolerable in elderly patients. However, the lack of conventional therapeutic options is also important in motivating the search for novel therapeutic approaches to pancreatic cancer. In the present case, the patient was diagnosed with advanced pancreatic cancer, and had a poor prognosis following surgery. Additionally, the patient was not able to tolerate the side-effects of traditional chemotherapy and radiotherapy. Subsequently, CIK cell immunotherapy alone was administered to the patient, achieving a beneficial effect.

Presently, immunotherapy has become the fourth treatment modality for malignant tumors, and may be a promising approach for pancreatic cancer (7–10). Certain studies have demonstrated that CIK cells are heterogeneous cell populations that express CD3 and CD56 as well as the activation receptor of NK cells (NKG2D), antigen, and possess MHC-unrestricted cytotoxicity toward pancreatic cancer but not toward normal targets (15,16). CIK cells are considered to be a type of antitumor effector cells, and proliferate rapidly *in vitro*, with stronger antitumor activity in a broad spectrum of tumors (9,17). In addition, CIK cells regulate and enhance cellular immune functions in patients with pancreatic cancer by secretion of cytokines, such as interferon-γ, and a number of chemokines, including RANTES, MIP-1α and MIP-1β (18,19). The application of CIK cells as adoptive immunotherapy is important in cancer treatment. The application of CIK cells as adoptive immunotherapy in solid tumor treatment has been frequently reported in the literature (10,20). In particular, Liu *et al* applied CIK cells to treat 74 cases of metastatic renal cell carcinoma patients in a randomized stage III clinical trial. The mean PFS was prolonged by 4 months and the mean overall survival time (mOS) was prolonged by 27 months compared with the control arm (10). Shi *et al* applied CIK cells to treat locally advanced gastric cancer patients and indicated that adjuvant immunotherapy with CIK cells prolonged disease-free survival (DFS) and significantly improved the mOS (21). However, there are few data concerned with applying CIK cells to treat pancreatic cancer in clinical practice. The present case provides a novel treatment option and further clinical practices are required to verify its efficacy.

In conclusion, we describe the case of a 77-year old female with pancreatic cancer who underwent surgery, although the bilateral margins were positive. After one month, imaging examination revealed that a low-density nodule had emerged at the resection margin. Considering the patient’s poor condition, low KPS scores and inability to tolerate the side-effects of chemotherapy and/or radiotherapy, the patient was treated with CIK cell immunotherapy. The patient achieved a PFS of >19 months, which was longer than that demonstrated by using chemotherapy and/or radiotherapy. In conclusion, CIK immunotherapy may be an effective treatment method for advanced pancreatic cancer, particularly for elderly patients.
